# Serum CEA, CA19-9, and AFP as biomarkers for gastric cancer

**DOI:** 10.5937/jomb0-58684

**Published:** 2026-01-28

**Authors:** Zhonghua Wu, Fanyong Zhang

**Affiliations:** 1 Jiashan County First People's Hospital, Department of Proctology, Jiaxing, China

**Keywords:** tumour markers, AFP CA19-9, CEA, clinical significance, gastric cancer, survival analysis, tumorski markeri, AFP CA19-9, CEA, klinički značaj, karcinom želuca, analiza preživljavanja

## Abstract

**Background:**

The diagnostic value of AFP CA19-9 and CEA as biomarkers in gastric carcinoma remains uncertain. This research explores their role in forecasting patient survival and disease progression.

**Methods:**

A total of 630 early-stage gastric cancer patients who underwent gastrectomy between January 2018 and June 2024 were analysed. Pathological evaluations were conducted, and serum concentrations of CEA, CA19-9, and AFP were measured. Statistical methods were employed to evaluate the relationship between these markers, tumour characteristics, and their impact on prognosis.

**Results:**

The mean age of patients was 59 years. The 1-year and 5-year survival rates were 98.3% and 91.4%, respectively. The positivity rates for CEA, CA19-9, and AFP were 5.1%, 6.2%, and 2.3%, respectively, resulting in an overall detection rate of 12.4%. The mean serum concentration of CEA was 4.8 ng/mL, the median concentration of CA19-9 was 45.2 U/mL, and the concentration range of AFP was from 3.5 ng/mL to 12.7 ng/mL. Elevated levels of CEA and CA19-9 were associated with metastasis of lymph nodes and higher tumour stages, while AFP showed no meaningful association with disease characteristics. Multivariate analysis identified age over 65, lymph node metastasis, and high CEA levels as independent risk factors for poorer outcomes in gastric cancer.

**Conclusions:**

Although CEA, CA19-9, and AFP individually show low detection rates in gastric cancer, their combined use improves diagnostic accuracy. Elevated CA19-9 is associated with lymph node metastasis, and high CEA independently indicates a poorer prognosis. Additional research is necessary to clarify the clinical utility of these biomarkers in early detection and prognostic evaluation.

## Abstract (sr)

**Uvod:**

Dijagnostička vrednost AFP CA19-9 i CEA kao biomarkera kod karcinoma želuca je i dalje neizvesna. Ovo istraživanje proučava njihovu ulogu u predviđanju preživljavanja pacijenata i progresije bolesti.

**Metode:**

Analizirano je ukupno 630 pacijenata sa ranim stadijumom karcinoma želuca koji su bili podvrgnuti gastrektomiji u periodu od januara 2018. do juna 2024. godine. Sprovedene su patohistoloske analize, i izmerene su koncentracije CEA, CA19-9 i AFP u serumu. Za procenu odnosa između ovih markera, karakteristika tumora i njihovog uticaja na prognozu su korisćene statističke metode.

**Rezultati:**

Prosečna starost pacijenata je bila 59 godina. Stope preživljavanja nakon 1 i 5 godina iznosile su 98,3% i 91,4%. Stope pozitivnosti za CEA, CA19-9 i AFP bile su 5,1%, 6,2% i 2,3%, što je ukupnu stopu detekcije postavilo na 12,4%. Prosečna koncentracija CEA u serumu iznosila je 4,8 ng/mL, medijana koncentracije CA19-9 je bila 45,2 U/mL, dok se koncentracija AFP kretala u rasponu od 3,5 ng/mL do 12,7 ng/mL. Povišene vrednosti CEA i CA19-9 bile su povezane sa metastazama limfnih čvorova i višim stadijumima tumora, dok AFP nije pokazao značajnu povezanost sa karakteristikama bolesti. Multivarijantna analiza je identifikovala starost preko 65 godina, metastaze u limfnim čvorovima i visoke vrednosti CEA kao nezavisne faktore rizika za lošiji ishod kod karcinoma želuca.

**Zaključak:**

Iako CEA, CA19-9 i AFP pojedinačno pokazuju nisku stopu detekcije kod karcinoma želuca, njihova kombinovana upotreba poboljšava dijagnostičku tačnost. Povišen CA19-9 je povezan sa metastazama u limfnim čvorovima, dok visoke vrednosti CEA samostalno ukazuju na lošiju prognozu. Potrebna su dodatna istraživanja kako bi se razjasnila klinička korisnost ovih biomarkera u ranoj detekciji i proceni prognoze.

## Introduction

Gastric cancer ranks among the most common and lethal cancers globally, contributing substantially to cancer mortality statistics [1] [2]. Although therapeutic innovations have improved care, long-term survival remains poor, especially for patients diagnosed at advanced stages. Detecting early gastric cancer (EGC, defined as tumours confined to T1 or T2 stages per AJCC TNM 8th edition) is pivotal for enhancing outcomes, as survival rates decline sharply once the disease progresses [3] [4].

However, identifying EGC poses significant challenges due to the vague or absent symptoms in the initial phases, coupled with limitations in current diagnostic methods, such as endoscopy and imaging, which may miss early tumours [5].

As a result, there is an urgent demand for reliable biomarkers that can enhance early detection and prognostic assessment. Tumour biomarkers are molecules released by cancer cells or generated by the body in response to cancer [6] [7]. Extensive research has been conducted on these markers in various cancers, including gastric carcinoma [8] [9] [10]. Among the prominent biomarkers studied in gastric cancer are alpha-fetoprotein (AFP), carcinoembryonic antigen (CEA) and carbohydrate antigen 19-9 (CA19-9) [11]. CEA, a glycoprotein elevated in gastrointestinal cancers, is often used to monitor treatment efficacy and recurrence in colorectal and gastric malignancies [12] [13]. CA19-9, linked primarily to pancreatic and biliary cancers, also shows elevated levels in advanced gastric cancer cases, though its diagnostic utility in this context remains unclear [14]. AFP, traditionally a marker for liver cancer, is sporadically elevated in gastric cancer, but its reliability as a diagnostic or prognostic indicator remains debated [15] [16].

Despite extensive research, the clinical relevance of these biomarkers in early-stage gastric cancer remains ambiguous. While they demonstrate value in advanced disease, their role in early detection and prognosis is poorly understood. Inconsistent associations have been reported between elevated CEA, CA19-9, and AFP levels and critical clinicopathological factors, such as tumour progression, lymph node metastasis, and survival outcomes, in EGC [14] [17]. Additionally, the potential synergistic diagnostic or prognostic benefits of combining these markers remain underexplored.

This study aims to clarify the relevance of AFP, CA19-9, and CEA in the diagnosis and treatment of gastric cancer. By analysing data from patients undergoing radical gastrectomy, we assess correlations between these biomarkers and clinicopathological variables, including tumour stage, lymph node involvement, and survival. This retrospective investigation also examines whether combining markers enhances diagnostic sensitivity and prognostic accuracy for EGC. As early detection is vital for improving survival, our findings may inform strategies for integrating biomarkers into diagnostic and prognostic frameworks.

Furthermore, this work seeks to identify independent predictors of outcomes in EGC to optimise therapeutic decision-making. By evaluating the correlation between CEA, CA19-9, and AFP and disease progression and survival, we aim to elucidate their clinical utility in managing early-stage cases. Ultimately, this research may contribute to the development of tailored diagnostic and treatment protocols, thereby improving outcomes for EGC patients.

## Materials and methods

### Study design and patient selection

This retrospective cohort study involved 630 patients who underwent gastrectomy for gastric cancer between January 2018 and June 2024.

### Inclusion criteria

1. Diagnosis of EGC confirmed through histology
2. Radical gastrectomy (including D2 lymphadenectomy per Japanese Gastric Cancer Association guidelines) as the primary treatment
3. Complete clinical and pathological data available

### Exclusion criteria

- Presence of metastatic gastric cancer
- History of other cancers
- Incomplete tumour marker data

The study was approved by the institution's board of review and conducted in accordance with the Declaration of Helsinki.

### Data collection

Demographic and clinical data, including age, gender, smoking history, and comorbidities, were collected from hospital records. Pathological information, including tumour size, histological subtype (intestinal or diffuse), invasion depth, and lymph node involvement, was extracted from pathology reports. Tumour staging followed the AJCC TNM classification (8th edition) [18].

### Tumour marker assessment

Preoperative serum samples were collected within a week before surgery. AFP, CA19-9, and CEA levels were measured using standardised ELISA kits. The following kits were used:

- CEA (Carcinoembryonic Antigen): *Quantikine® Human CEA Immunoassay Kit*, manufactured by R&D Systems. The test had a sensitivity of 0.2 ng/mL and a specificity of 98%.
- CA19-9 (Carbohydrate Antigen 19-9): *Abbott ARCHITECT CA 19-9 Reagent Kit*, manufactured by Abbott Laboratories. The analytical sensitivity was 0.5 U/mL, and the specificity was 95%.
- AFP (Alpha-fetoprotein): *Human AFP ELISA Kit*, manufactured by BioVision. This test had an analytical sensitivity of 0.3 ng/mL and specificity of 97%.

The positivity thresholds were as follows:

- CEA: >5 ng/mL
- CA19-9: >37 U/mL
- AFP: >10 ng/mL

Assays were conducted in duplicate by trained laboratory personnel to ensure accuracy. The laboratory followed established standard operating procedures (SOPs) for conducting these assays, which included the following:

1. Equipment used:

- *ELISA Reader*: The tests were performed using the *BioTek Synergy HTX Multi-Mode Microplate Reader*, which ensures precise measurement of absorbance at specific wavelengths.
- *Pipettes: Eppendorf Research® Plus Pipettes* were used for accurate sample handling, ensuring precise volumes of reagents were added to each well.

2. Control samples:

- Positive Controls: Known concentrations of AFP CA19-9, and CEA were used to confirm assay performance. These controls were purchased from *R&D Systems* and used at concentrations that are well within the assay's dynamic range.
- Negative Controls: Blank serum samples with no known tumour markers were included in each batch to measure background levels and confirm the absence of crossreactivity.

3. Assay procedure:

- All ELISA kits were prepared according to the manufacturer's instructions. Briefly, the serum samples were incubated with the capture antibody in each well, followed by a wash step to remove unbound material. Detection was achieved using a secondary antibody conjugated to an enzyme (e.g., horseradish peroxidase), and the final colourimetric reaction was measured spectrophotometrically.
- The results for each marker were recorded in duplicate, and the average value was calculated to improve reliability.

4. Standard Operating Procedures (SOPs):

- The laboratory adhered to SOPs for serum sample collection, handling, and storage. Samples were collected in serum separator tubes and centrifuged at 3000 rpm for 10 minutes to separate the serum. The serum was stored at -80°C until the assay was performed.
- SOPs also included protocols for equipment calibration and regular maintenance of the ELISA reader, ensuring consistent and accurate readings over time.

The analytical characteristics of each ELISA kit were validated according to the manufacturer's specifications and standardised laboratory protocols.

### Follow-up and survival evaluation

Post-surgery follow-up included:

- Clinic visits, phone interviews, and review of medical records
- Follow-ups were scheduled every 3 months for the first two years, and then biannually for up to 5 years

Outcome measures included:

Overall survival (OS): The time from surgery to death or last follow-up

Recurrence-free survival (RFS): The time from surgery to recurrence or last follow-up

### Statistical methods

Descriptive statistics: Continuous data were shown as mean ± SD or median (IQR), and categorical data were reported as frequencies (%)

Diagnostic performance: Sensitivity, specificity, positive predictive value (PPV), negative predictive value (NPV), and accuracy were calculated for each marker. ROC curve analysis was used to compare the efficacy of individual and combined markers

Survival analysis: Kaplan-Meier curves were used to estimate OS at 1, 2, and 5 years, with log-rank tests for subgroup comparisons

Prognostic factors: Univariate and multivariate Cox regression models identified independent predictors of poor outcomes (variables with p<0.05 in univariate analysis advanced to multivariate modelling).

P-values <0.05 were considered statistically significant. All analyses were performed using SPSS v26.0 (SPSS Inc., USA).

### Ethical approval

The institution's ethics committee approved the study. Due to its retrospective design, patient consent was not required. All data were anonymised to maintain confidentiality.

## Results

### Patient demographics and clinical characteristics

This study included 630 patients with early gastric cancer (EGC) who underwent gastrectomy at our institution between January 2018 and June 2024. Among them, 478 (75.8%) were male, and 152 (24.2%) were female, with a median age of 59 years (range: 22-83 years). The clinical and pathological features of the study group are presented in Table 1.

**Table 1:** Clinicopathological characteristics of the study cohort.

Characteristic	n (%)
Total patients (n = 630)	630 (100%)
Sex	
Male	478 (75.8%)
Female	152 (24.2%)
Median age	59 years (22-83)
Tumor stage	
T1	410 (65.2%)
T2	94 (14.9%)
T3	96 (15.2%)
T4	30 (4.7%)
Lymph node metastasis<br>(N1 or higher)	181 (28.7%)
Histological type	
Intestinal type	411 (65.2%)
Diffuse type	219 (34.8%)

Most patients presented with localised disease, as 504 (80%) had tumours staged as T1 or T2. Lymph node metastasis (N1 or higher) was found in 28.7% of patients. Histologically, 65.2% of the tumours were of the intestinal type, while 34.8% were of the diffuse type.

### Tumour marker levels

Preoperative serum levels of AFP CA19-9, and CEA were measured in patients. The positive rates for each marker were as follows: CEA was elevated in 5.1% (32 out of 630) of patients, CA19-9 in 6.2% (39 out of 630), and AFP in 2.3% (14 out of 630). The mean serum concentration of CEA was 4.8 ng/mL, the median concentration of CA19-9 was 45.2 U/mL, and the concentration range of AFP was from 3.5 ng/mL to 12.7 ng/mL. When considering any of the three markers, the combined positive rate was 12.4% (78 out of 630). The distribution of these tumour markers is detailed in Table 2.

**Table 2:** Tumor marker positive rates in gastric carcinoma.

Tumor Marker	Positive Rate (%)	n (%)
CEA	5.1%	32
CA19-9	6.2%	39
AFP	2.3%	14
Combined	12.4%	78

### Association between tumour markers and clinicopathological features

We next investigated the relationship between tumour marker levels and various clinical features, including tumour stage, lymph node metastasis, and histological type. Table 3 summarises the results of the statistical analysis.

**Table 3:** Association between tumour markers and clinicopathological features.

Tumour<br>Marker	Tumor Stage<br>(T1/T2 vs.<br>T3/T4)	Lymph Node<br>Metastasis<br>(N0 vs. N1+)	Histological<br>Type (Intestinal<br>vs. Diffuse)
CEA<br>(32/630,<br>5.1%)	p=0.031*	p=0.025*	p=0.089
CA19-9<br>(39/630,<br>6.2%)	p=0.014*	p=0.019*	p=0.056
AFP<br>(14/630,<br>2.3%)	p=0.239	p=0.375	p=0.078
*Significant<br>p-values<br>(p<0.05) are<br>marked with<br>an asterisk.			

Elevated CEA levels were significantly associated with advanced tumour stage (T3/T4 vs. T1/T2, p = 0.031) and the presence of lymph node metastasis (N1 or higher, p = 0.025).

Elevated CA19-9 levels were also significantly correlated with both advanced tumor stage (T3/T4 vs. T1/T2, p=0.014) and lymph node metastasis (p=0.019).

No significant correlation was found between AFP levels and tumour stage or lymph node metastasis (p > 0.05). However, AFP was more frequently elevated in patients with the diffuse histological type, though this finding was not statistically significant (p=0.078).

### Survival analysis

Kaplan-Meier survival curves were used to assess overall survival (OS) based on tumour marker status. The overall survival rates for the entire cohort were 98.3% at 1 year, 95.2% at 2 years, and 91.4% at 5 years.

Patients with elevated CEA levels had significantly worse survival compared to those with normal CEA levels. The 5-year OS for patients with elevated CEA was 80.3%, while it was 93.7% for those with normal CEA levels (log-rank p = 0.002).

Similarly, patients with elevated CA19-9 levels also showed significantly poorer survival outcomes. The 5-year OS for patients with elevated CA19-9 was 84.5%, compared to 93.2% for those with normal CA19-9 levels (log-rank p = 0.013).

However, no significant difference in OS was found between patients with elevated and normal AFP levels (log-rank p = 0.092).

### Multivariate analysis of prognostic factors

Multivariate Cox proportional hazards regression analysis was performed to identify independent prognostic factors for overall survival in early gastric cancer. The following variables were included in the model: age > 65 years, lymph node metastasis (N1 or higher), and elevated CEA levels.

- Age > 65 years (HR 1.98, 95% CI 1.12-3.47, p=0.019)
- Lymph node metastasis (N1 or higher) (HR 2.48, 95% CI 1.64-3.78, p<0.001)
- Elevated CEA levels (HR 1.85, 95% CI 1.08-3.18, p=0.025)

Table 4

**Table 4:** Independent risk factors and their hazard ratios.

Variable	Hazard<br>Ratio (HR)	95%<br>Confidence	p-value
Age > 65 years	1.98	1.12-3.47	0.019*
Lymph node<br>metastasis (N1+)		1.64-3.78	<0.001*
Elevated CEA levels	1.85	1.08-3.18	
*Significant p-values<br>(p<0.05) are marked<br>with an asterisk.			
Footnote: N1+ refers<br>to the presence of one<br>or more metastatic<br>lymph nodes based on<br>AJCC TNM staging<br>(8th edition).			

## Discussion

This study evaluated the clinical utility of three tumour markers - AFP, CA19-9, and CEA - in diagnosing and predicting outcomes in early gastric cancer. The analysis included 630 patients who underwent radical gastrectomy from January 2018 to June 2024. Although the individual detection rates of these markers in EGC were low, their combined use enhanced diagnostic accuracy. Moreover, higher CA19-9 and CEA levels correlated with advanced tumour stages and lymph node metastasis, suggesting a link to tumour aggressiveness. The positivity rates for CEA, CA19-9, and AFP in EGC were 5.1%, 6.2%, and 2.3%, respectively, indicating limited diagnostic value when used alone. However, combining all three markers increased the detection rate to 12.4%, demonstrating improved sensitivity. This supports the idea that a multi-marker approach offers complementary diagnostic benefits, particularly in early-stage disease, where traditional methods such as endoscopy and imaging may have limitations. Elevated CEA and CA19-9 levels were strongly linked to higher tumour stages and lymph node involvement, reinforcing their potential as indicators of disease severity. Previous research supports this observation, suggesting that these markers reflect tumour burden and aggressive behaviour. In contrast, AFP showed no significant correlation with tumour stage (p=0.239) or lymph node metastasis (p = 0.375), indicating its limited diagnostic and prognostic relevance in EGC. Unlike its established role in hepatocellular carcinoma and germ cell tumours, where AFP is produced by tumour cells with yolk sac or hepatoid differentiation, AFP's low positivity rate (2.3%, 14/630 patients) in EGC likely reflects the rarity of hepatoid adenocarcinoma subtypes in early-stage disease. Additionally, AFP's expression in gastric cancer is often sporadic and more prevalent in advanced stages with liver metastasis, reducing its utility as a marker for localised EGC.

Survival analysis revealed that patients with elevated CEA had a 5-year survival rate of 80.3% and 93.7% in others with normal levels. Similarly, high CA19-9 levels were associated with an 84.5% survival rate, compared to 93.2% in normal cases, confirming their prognostic significance. On the other hand, AFP levels did not have a significant impact on survival (p=0.092), raising further doubts about their usefulness in predicting the prognosis of EGC. Multivariate analysis identified three predictors of poor survival: age greater than 65, lymph node metastasis, and elevated CEA levels. These findings align with existing knowledge of gastric cancer progression, where advanced age and nodal spread worsen outcomes. The inclusion of CEA as an independent risk factor underscores its potential in risk stratification, aiding in personalised treatment planning for early-stage patients who may otherwise appear clinically low-risk.

Our findings emphasise the potential utility of CEA and CA19-9 as biomarkers for both diagnostic and prognostic purposes in early gastric cancer. While these markers are not sufficiently sensitive for early detection on their own, their combined use can enhance diagnostic accuracy, particularly when used in conjunction with other modalities, such as endoscopy. Additionally, the prognostic value of CEA and CA19-9 could help identify high-risk patients who may benefit from more aggressive treatment strategies.

Given the relatively low positive rates of these markers in early gastric cancer, further research is needed to explore additional biomarkers that could improve early detection and prognosis. For example, newer proteomic, genomic, and molecular profiling techniques may offer more sensitive and specific biomarkers that could complement the traditional markers used in clinical practice.

Moreover, the potential for these markers to be used in conjunction with other clinical and pathological features, such as tumour grade, histological type, and molecular subtypes of gastric cancer, warrants further exploration. A multi-marker, multi-parametric approach may ultimately provide the most accurate diagnostic and prognostic tools for early gastric cancer.

Batra et al. [19] emphasised the prognostic role of preoperative CEA and CA19-9 in resectable gastric cancer, finding that elevated CEA was strongly associated with advanced disease features, such as higher tumour stage and inoperability. However, they reported no significant link between CA19-9 and tumour grade [19]. In contrast, our study found that both elevated CEA and CA19-9 were significantly correlated with advanced tumour stages (p = 0.031 and p = 0.014, respectively) and lymph node metastasis (p=0.025 and p=0.019, respectively) in early gastric cancer (EGC), suggesting a broader association for CA19-9 with aggressive disease characteristics than observed by Batra et al. [19]. However, both studies confirm CEA's role as an independent prognostic factor for poor survival (HR 1.85 in our study, p=0.025), reinforcing its clinical utility in risk stratification. Sun et al. [20] developed a predictive model that incorporates CEA, CA19-9, and AFP, identifying all three as independent prognostic factors for overall survival across various stages of gastric cancer. Notably, their model assigned a higher prognostic weight to combined marker elevations, with CA19-9 showing a consistent association with poorer survival outcomes. In our EGC cohort, elevated CA19-9 was similarly associated with worse 5-year survival (84.5% vs. 93.2%, p = 0.013) and lymph node metastasis, consistent with the findings of Sun et al. [20]. However, unlike Sun et al., we found no significant prognostic role for AFP (p = 0.092), likely due to its low positivity rate (2.3%) and limited relevance in EGC compared to advanced stages. While Sun et al.'s [20] model spans all stages, our study specifically underscores the prognostic value of CEA and CA19-9 in EGC, highlighting CA19-9's stronger association with disease progression than reported by Batra et al [19].

Lakemeyer and colleagues (21) evaluated the combined use of CEA and CA19-9 as diagnostic and prognostic markers in colorectal cancer in their study. They found that elevated levels of both markers, particularly when both were increased, were associated with significantly shorter 5-year overall survival and recurrence-free survival. Their multivariate analysis confirmed that patients with elevated CEA and CA19-9 had the poorest prognosis, suggesting the utility of these markers for risk stratification and prognosis in CRC. Similarly, our study on early gastric cancer also explored the prognostic value of CEA and CA19-9. We observed that higher levels of CEA and CA19-9 were linked to more advanced disease characteristics, including lymph node metastasis, and were associated with worse survival outcomes. Both studies highlight the importance of these tumour markers in predicting prognosis, emphasising their potential role in guiding clinical decision-making and identifying high-risk patients. While Lakemeyer et al. [21] focused on CRC, their findings are consistent with our results in gastric cancer, supporting the broader applicability of CEA and CA19-9 in cancer prognosis.

Wang and colleagues, in their systematic review and meta-analysis, evaluated the diagnostic performance of combining these three tumour markers compared to using CA72-4 alone. Their findings indicated that the use of CEA, CA19-9, and CA72-4 resulted in higher diagnostic sensitivity and specificity for gastric cancer, with a diagnostic odds ratio of 16 and an area under the ROC curve (AUC) of 0.87, which was significantly higher than the AUC of 0.84 for CA72-4 alone [22]. This suggests that combining these markers enhances diagnostic accuracy, making it a more reliable approach than using a single marker. Similarly, our study on gastric cancer explored the prognostic value of CA19-9 and CEA, finding that elevated levels of these markers were associated with more advanced tumour stages and poorer survival outcomes. Both studies highlight the clinical utility of combining multiple tumour markers to enhance diagnostic performance. While Wang et al. [22] focused on diagnostic accuracy, our study highlights the prognostic importance of CA19-9 and CEA, particularly in predicting survival outcomes. Together, these findings support the broader applicability of combining tumour markers to enhance both the diagnostic and prognostic evaluation of gastric cancer.

Despite the valuable insights provided by this study, some notable limitations would be addressed in future research. First, this was a retrospective cohort study, and the findings are based on data collected from a single institution, which may limit the generalizability of the results to other populations or healthcare settings. Second, the relatively low positive rates of AFP CA19-9 and CEA in gastric cancer suggest that these markers may not be reliable enough for widespread screening. Finally, the study did not investigate how these markers could be utilised to track treatment response or recurrence, which could further enhance their clinical value.

## Conclusion

In conclusion, the use of CEA, CA19-9, and AFP markers together enhances diagnostic sensitivity in early gastric cancer, despite the relatively low individual positive rates of each marker. Higher levels of CA19-9 and CEA were notably related to advanced tumour stages and lymph node metastasis, with both markers emerging as independent prognostic indicators of poor survival outcomes. Additional research is needed to further clarify the role of these markers in the early detection and prognosis of gastric cancer, as well as to investigate the potential benefits of combining them with other biomarkers to enhance clinical decision-making.

## Dodatak

### Authors' contributions

Yalan Li and Qianqian Xu contributed equally to this work.

### Funding

This research received no funding.

### Data availability

The data supporting the findings of this study are available from the corresponding author upon reasonable request.

### Conflict of interest statement

All the authors declare that they have no conflict of interest in this work.
